# Medial Malleolus Triple‐Plane Osteotomy With Autologous Iliac Bone Grafting for the Treatment of Talar Cysts

**DOI:** 10.1111/os.70308

**Published:** 2026-04-09

**Authors:** Yanbin Teng, Yan Zhang, Longxin An, Jie Zhao, Maoyuan Xin, Xiaoming Yang

**Affiliations:** ^1^ Department of Orthopedics and Trauma Weifang People's Hospital (First Affiliated Hospital of Shandong Second Medical University) Weifang China; ^2^ Department of Nephrology Weifang People's Hospital (First Affiliated Hospital of Shandong Second Medical University) Weifang China; ^3^ Shandong Second Medical University Weifang China

**Keywords:** autologous iliac bone grafting, case series, medial malleolus triple‐plane osteotomy technique, talar cysts

## Abstract

**Objectives:**

Talar cysts, a common manifestation of osteochondral lesions of the talus (OLT), often result from trauma and cause significant pain and dysfunction. Cysts > 10 mm require grafting, and autologous iliac bone is an ideal graft source. However, accessing posteromedial lesions remains challenging: oblique osteotomies are associated with high osteoarthritis rates (up to 50%) and delayed union, while bi plane chevron osteotomies have reported malunion rates as high as 30%. To address these limitations, this study investigated the effectiveness of medial malleolus triple plane osteotomy combined with autologous iliac bone grafting for large talar cysts.

**Methods:**

This retrospective case series included patients with talar cysts larger than 10 mm in diameter who were treated in our Hospital between February 2021 and March 2023. Patients underwent medial malleolus triple‐plane osteotomy with autologous iliac bone grafting. Postoperative outcomes were assessed, including neurological complications, skin healing, radiological assessment of fracture healing, graft fusion, joint space evaluation, American Orthopedic Foot and Ankle Society Ankle‐Hindfoot Score (AOFAS‐AHS), Kaikkonen functional scores, and Visual Analogue Scale (VAS) scores. Statistical analyses were performed using paired *t*‐tests to compare preoperative and final follow‐up scores.

**Results:**

A total of nine patients (mean age 34.20 ± 9.23 years; seven males) were included, which had a follow‐up period of 23.00 ± 7.80 months. Primary wound healing was achieved in all cases, with no neurovascular injuries reported. Postoperative X‐rays demonstrated that the osteotomy lines became indistinct at 3 months, disappeared progressively by 6 months, and were fully healed by 12 months, at which point the internal fixation was removed. The grafted talar region exhibited sclerosis at 3 months, progressive assimilation with surrounding bone density at 6 months, and subtle visibility of the graft site at 12 months. At final follow‐up, compared to preoperative values, patients had significantly improved VAS scores (*p* < 0.05), AOFAS‐AHS scores (92.37 ± 2.09 vs. 59.39 ± 6.31, *p* < 0.05), and Kaikkonen functional scores (89.11 ± 3.11 vs. 60.23 ± 2.79, *p* < 0.05).

**Conclusions:**

Medial malleolus triple‐plane osteotomy and autologous iliac bone grafting might be effective in treating talar cysts in this series of patients. Patients experienced less pain and improved functional scores at the final follow‐up with no neurovascular injuries.

## Graphical abstract

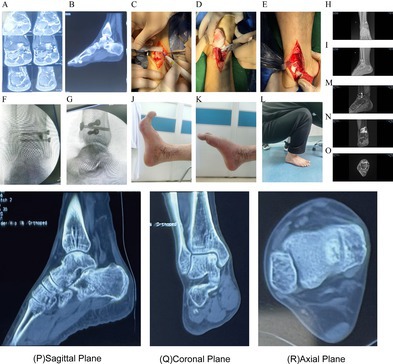

**Abbreviations**
AOFAS‐AHS: American Orthopedic Foot and Ankle Society Ankle‐Hindfoot Score
CT: computed tomography
MRI: magnetic resonance imaging
OLT: osteochondral lesions of the talus
SD: standard deviation
VAS: Visual Analogue Scale

## 1 Introduction

Talar cysts represent a manifestation of osteochondral lesions of the talus (OLT), with trauma being the primary etiological factor [1]. Approximately 50%–70% of ankle sprains or fractures may be associated with OLT [2]. Among these cases, a significant proportion exhibit talar cysts of varying sizes. While the detailed etiology of talar cysts is unclear, when trauma occurs due to an event such as an ankle sprain, the talus can impact on the distal tibial plafond and cause microfractures in the cartilage and subchondral bone plate. The microfractures can then fill with synovial fluid when under pressure due to weight bearing, leading to osteonecrosis which can expand to form a cyst [3]. Therefore, most cases present with pain 6–12 months after the initial trauma [3].

Treatment for OLT can include conservative management for around 6 months, but talar cysts often require surgery to relieve pain and prevent the risk of fracture [1, 3]. Small, superficial cysts can be managed with arthroscopic bone marrow stimulation or open reduction and internal fixation [4]. Microfracture techniques, autologous cartilage transplantation, allogeneic cartilage transplantation, bone‐periosteum grafting, and structural allografts of hemi‐talus bones are among the various methods used [1, 2, 5, 6]. The microfracture technique involves drilling into the cyst wall after debridement to stimulate the formation of fibrocartilage. This approach aims to increase local blood flow within the talus to the lesion site, forming a blood clot that promotes fibrous repair by generating variable amounts of type II collagen [5]. However, several studies have indicated that this method is effective only for lesions smaller than 1.5 cm^2^, and its efficacy diminishes for larger lesions [7]. Cysts with a diameter exceeding 10 mm and a depth greater than 6 mm require grafting to fill the defect [8].

Graft materials include autologous or allogeneic cartilage, bone‐periosteum composites, chondrocytes, and tissue‐engineered stem cell constructs and may depend upon the characteristics of the lesion [9, 10]. Autologous cartilage transplantation from the patellar trochlea of the knee joint carries the risk of poor knee function postoperatively, with reported complications ranging from 2% to 50% [11]. Allogeneic cartilage transplantation has not been widely adopted due to limitations in donor availability, donor quality, risks of immune rejection, bone resorption, and economic constraints [12, 13]. Autologous iliac bone grafting is common in a wide range of orthopedic and trauma surgeries [14]. For treatment of a large cyst, it may have distinct advantages: the iliac bone provides a substantial volume of graft material, making it suitable for large talar cysts regardless of lesion size. It has an optimal balance of hardness and plasticity, offering both structural support and adaptability for shaping into various geometries, and an autologous graft eliminates risks of immune rejection or disease transmission and has a low incidence of donor site complications [1, 15].

Alongside grafting various osteotomy techniques, such as triple‐plane osteotomy, chevron osteotomy, trapdoor osteotomy, and oblique osteotomy, have been employed for access and management [16]. Medial malleolar osteotomy is a common approach among these techniques [17]. However, the evidence for the most suitable technique is lacking and much depends on the preference and experience of the clinical team [18]. In our experience, triple‐plane osteotomy has several advantages, including a broad surgical field, short learning curve, straightforward reduction, and stable postoperative fixation, with rapid joint recovery.

Considering the ongoing debate about the preferred graft and osteotomy techniques for treating large talar cysts, we felt it important to present our experience of using medial malleolus triple‐plane osteotomy combined with autologous iliac bone grafting. This will provide evidence of the effectiveness of this approach for treating large talar cysts. Therefore, the aim of this study was to retrospectively analyze nine cases of talar cysts treated in our department from February 2021 to March 2023. Therefore, the purposes of this study were: (i) to describe the surgical technique and feasibility of medial malleolus triple‐plane osteotomy combined with autologous iliac bone grafting for the treatment of large talar cysts; (ii) to evaluate the clinical and radiographic outcomes of this procedure in a series of nine patients treated between February 2021 and March 2023; and (iii) to assess the safety and complication profile of this combined procedure.

## 2 Methods

### 2.1 Study Design and Patients

This retrospective case series included patients who underwent treatment for talar cysts at our hospital between February 2021 and March 2023. The inclusion criteria were as follows: (1) diagnosis of a talar cyst confirmed by preoperative imaging (X‐ray and/or computed tomography [CT]); (2) cyst diameter greater than 10 mm; (3) presence of significant clinical symptoms (e.g., pain, functional limitation) affecting daily activities or sports, warranting surgical intervention; and (4) surgical management via medial malleolus triple‐plane osteotomy combined with autologous iliac bone grafting. Exclusion criteria comprised: (1) active infection in the ipsilateral ankle joint; (2) concurrent ankle joint instability; (3) ankle joint ankylosis or severe arthrosis; and (4) significant malalignment of the lower limb mechanical axis. The study was approved by the Institutional Review Board (or Ethics Committee) of our hospital(IRB/IEC No. KYLL20241220‐2). Given the retrospective nature of the analysis, the requirement for written informed consent was waived.

### 2.2 Typical Treatment

#### 2.2.1 Preoperative Preparation

Routine preoperative evaluations included complete blood count, biochemical markers, erythrocyte sedimentation rate, and C‐reactive protein levels. Radiological assessments comprised standard anteroposterior and lateral X‐rays, ankle CT scans, and when necessary, magnetic resonance imaging (MRI) of the ankle. Specialized clinical examinations were performed to exclude ankle joint instability, evaluate the condition of the surrounding skin, and measure the range of motion of the ankle joint.

#### 2.2.2 Surgical Procedure

(1) *Incision and exposure*: A medial incision was made adjacent to the tibialis anterior tendon on the medial aspect of the ankle. Layer‐by‐layer dissection was performed to expose the entire medial malleolus and the anterior ankle joint. Throughout the procedure, care was taken to protect the saphenous nerve, the great saphenous vein, and its branches. The continuity of the deltoid ligament was palpated to confirm its integrity. (2) *Triple‐plane osteotomy*: Sagittal Plane: Using a thin, calibrated oscillating saw, osteotomy was initiated along the tibial midline, perpendicular to the tibial articular surface. The osteotomy height was approximately 3 cm, with a depth reaching half the anteroposterior diameter of the tibia. If the lesion was located on the medial‐most part of the talus, the osteotomy line was shifted medially to the medial one‐third of the tibial articular surface. (3) *Coronal plane*: The osteotomy began posterior to the anterior colliculus of the medial malleolus. The cut was aligned with the midline of the medial malleolus, matching the height of the sagittal osteotomy, with a depth reaching half the mediolateral diameter of the tibia. (4) *Horizontal plane*: The horizontal osteotomy was aligned perpendicular to the sagittal and coronal osteotomy lines, connecting the cortical bones between these two cuts. The depth of the horizontal osteotomy extended to half the diameter of the tibia. (5) Upon completing the osteotomy, sharp dissection was performed with a bone elevator or a No. 20 blade to separate the osteotomy block down to the joint surface while preserving the posterior structures of the medial malleolus. The osteotomy block was flipped to expose the talar lesion. (6) *Lesion debridement*: A probe or vascular clamp was used to locate the soft cartilage at the top of the cyst. Necrotic tissue was curetted, and the cyst wall was refreshed. The lesion was shaped as uniformly as possible to facilitate subsequent grafting. (7) *Iliac bone grafting*: After measuring the diameter and depth of the lesion with a depth gage, bone was harvested from the anterior superior iliac spine. The graft dimensions slightly exceeded the lesion size. A micro‐burr was used to shape the graft to an appropriate size, and it was press‐fit into the lesion. (8) *Repositioning and internal fixation*: The osteotomy block was repositioned and temporarily fixed with clamps. Two 4.0‐mm cannulated screws were inserted through the medial malleolus along the coronal plane, while one 4.0‐mm cannulated screw was inserted from anterior to posterior along the sagittal plane. After confirming optimal positioning under fluoroscopy, the wound was sutured in layers.

#### 2.2.3 Postoperative Management

A single dose of antibiotics was administered postoperatively to prevent infection, and measures were taken to reduce swelling and manage pain. The ankle joint was immobilized with an external orthosis. Sutures were removed 2 weeks postoperatively, after which patients were instructed to remove the orthosis daily for passive ankle joint exercises, focusing solely on flexion and extension movements. At 6 weeks postoperatively, patients began toe‐touch weight‐bearing exercises using double crutches. At 3 months postoperatively, following X‐ray re‐evaluation, patients were instructed to gradually transition to full weight‐bearing. Daily activities and physical exercise were resumed progressively.

### 2.3 Data Collection and Definitions

The baseline data including patient gender, age, history of trauma to the ankle, and sports activity were collected. The clinical data, including the routine preoperative evaluations described above, operative duration, and intraoperative techniques for each case were also recorded. Specified that graft fusion was assessed via serial X‐rays, with acknowledgment of CT as a superior but not routinely performed modality in this retrospective series. Postoperative X‐rays were taken at 3, 6, and 12 months, and the range of motion of the ankle joint was assessed during each follow‐up. American Orthopedic Foot and Ankle Society Ankle‐Hindfoot Score (AOFAS‐AHS) and the Kaikkonen functional score for ankle injuries were used to measure joint function [19] and Visual Analogue Scale (VAS) was used to measure the degree of pain experienced by the patients [20].

### 2.4 Statistical Analysis

Comparisons were made between preoperative and final follow‐up scores for the AOFAS‐AHS, Kaikkonen functional score, and VAS. Paired *t*‐tests were used to compare preoperative and final follow‐up scores, as the data were confirmed to be normally distributed by the Shapiro–Wilk test. Statistical analysis was performed using R v4.4.1, along with Zstats v1.0 (http://www.zstats.net). Normally distributed data were expressed as the mean ± standard deviation (SD). Statistical significance was defined as two‐sided *p* < 0.05.

## 3 Result

### 3.1 Basic Characteristics

A total of nine cases (nine ankles) were included, comprising seven males and two females, with a mean age of 34.20 ± 9.23 years. Among these, six cases had a history of significant trauma, while three patients did not recall any clear history of trauma. Five cases reported regular participation in sports activities, although the types of sports varied. One patient was a secondary school physical education teacher. Most patients experienced symptoms of pain for over 1 year before seeking medical attention.

### 3.2 Outcomes

All nine patients were successfully followed up for a mean duration of 23.00 ± 7.80 months. Primary wound healing was achieved in all cases, with no instances of neurovascular injury. Postoperative X‐rays showed that the osteotomy lines began to blur at 3 months, gradually disappeared by 6 months, and fully healed by 12 months, at which point the internal fixation was removed. In the talar graft area, sclerosis was observed at 3 months, with gradual assimilation of the graft density into the surrounding bone by 6 months. By 12 months, the graft area was faintly visible.

A statistically significant improvement in VAS scores was observed at the final follow‐up compared to preoperative scores (*p* < 0.05). The AOFAS‐AHS score improved significantly from 59.39 ± 6.31 preoperatively to 92.37 ± 2.09 at the final follow‐up (*p* < 0.05). Similarly, the Kaikkonen functional score for ankle injuries increased from 60.23 ± 2.79 preoperatively to 89.11 ± 3.11 at the final follow‐up, with a statistically significant difference (*p* < 0.05).

### 3.3 A Typical Case

**FIGURE 1:**
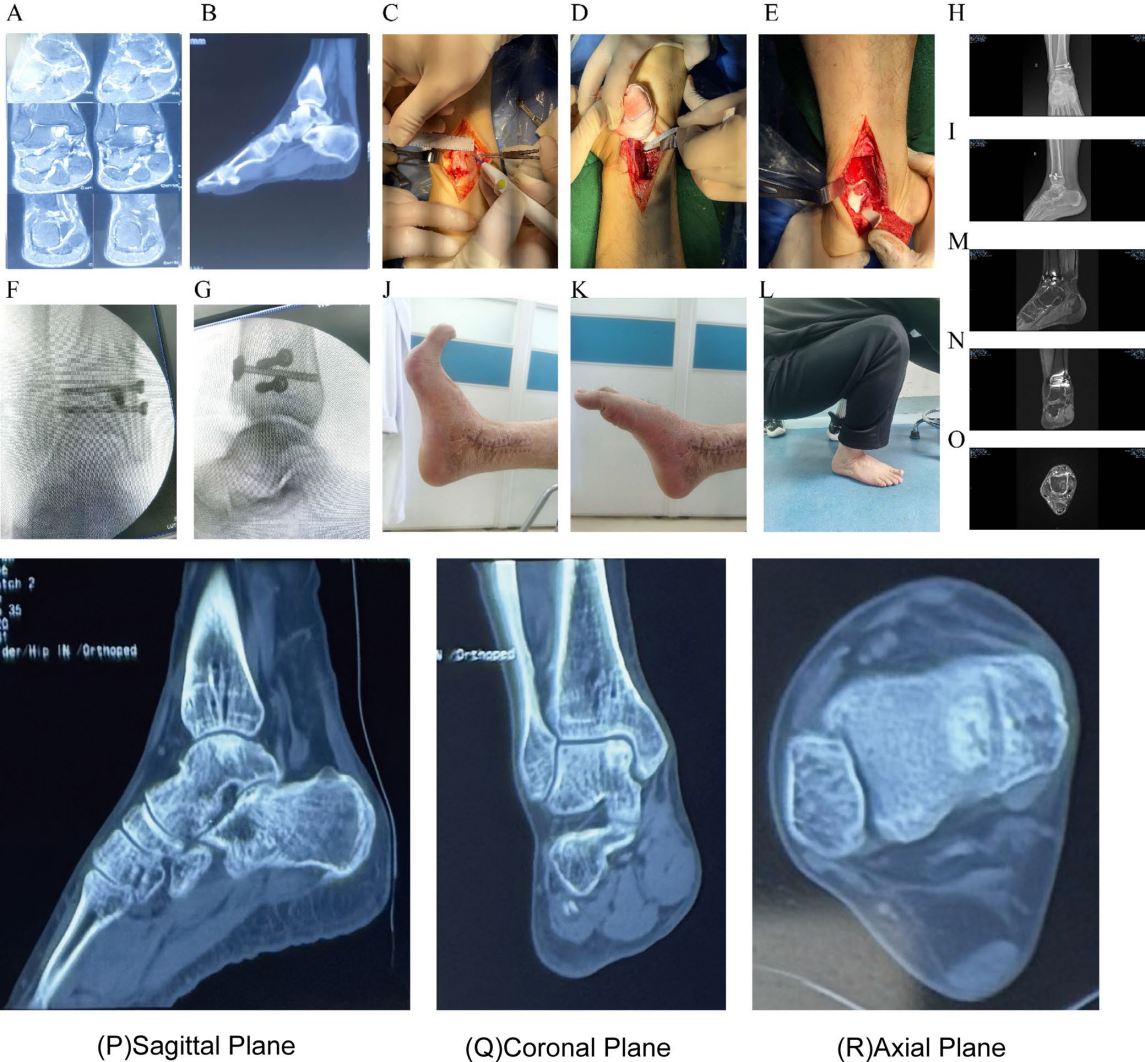
Images from a typical case of a large talar cyst. (A) Preoperative magnetic resonance image (MRI); (B) preoperative computed tomography (CT) image; (C–E) intraoperative surgical images, measurement of osteotomy range (C), triplanar osteotomy of the medial ankle (D), revealing and clearing the talus cyst (E); (F and G) intraoperative perspectives of the right ankle joint; (H and I) reexamination of the right ankle arthrosis 3 months after surgery; (J–L) re‐examination of ankle dorsiflexion, flexion function, and activity of ankle joint in squatting position with weight bearing 6 months after surgery; (M and N) MRI of the right ankle 15 months after surgery. Sagittal view (M), coronal view (N), all the MRI levels of the right ankle joints showed that the talus cyst was well repaired, and there was no obvious inflammatory reaction around it (O); Postoperative CT follow‐up data (P–R) at 30 months, it can be observed that the grafted bone has achieved complete fusion.

A 45‐year‐old male patient was admitted to the hospital with “right ankle pain that worsened 4 years after injury.” Four years ago, the patient sprained his right ankle while walking and felt pain immediately. The ankle gradually swelled, but there was no loss of movement or skin damage, and no special treatment was given. Later, the patient felt increasing pain after moving his right ankle. More than half a month prior to attending our hospital, he went to a local hospital for a CT examination, which showed no traumatic changes in the bone of the right ankle. There was evidence of a low‐density cystic lesion of the right talus, not excepting an adjacent joint cyst. No special treatment was performed. For further diagnosis and treatment, he then attended the outpatient department of our hospital. Based on his medical history and related examinations, the patient was admitted to the hospital with “right talus cyst and right talus cartilage injury” (Figure 1A,B). Other than the ankle pain since the injury, the patient was in good health and good spirits without any significant change in weight.

Medial ankle osteotomy under tracheal intubation and general anesthesia was performed (Figure 1C–G), with talus lesion excision, while an iliac bone excision was used as the graft. The operation was successful; symptomatic treatment such as swelling reduction, pain relief, and thrombosis prevention were given after surgery. A rehabilitation physician regularly guided the patient in functional rehabilitation exercises, and the patient was discharged successfully. After 2 weeks, 1 month, 2 months, 3 months, 6 months, 15 months, and 30 months, the wound healing and functional exercise of the patients were observed (Figure 1H–O). The patient returned to our hospital 15 months after the operation for removal of the internal fixation device of the right ankle. The operation was successful, and symptomatic treatment such as swelling and pain relief was given after the operation. The patient recovered well and was discharged from the hospital. Postoperative CT follow‐up data (Figure 1P–R) at 30 months indicated that the grafted bone has achieved complete fusion.

### 3.4 Another Typical Case

**FIGURE 2:**
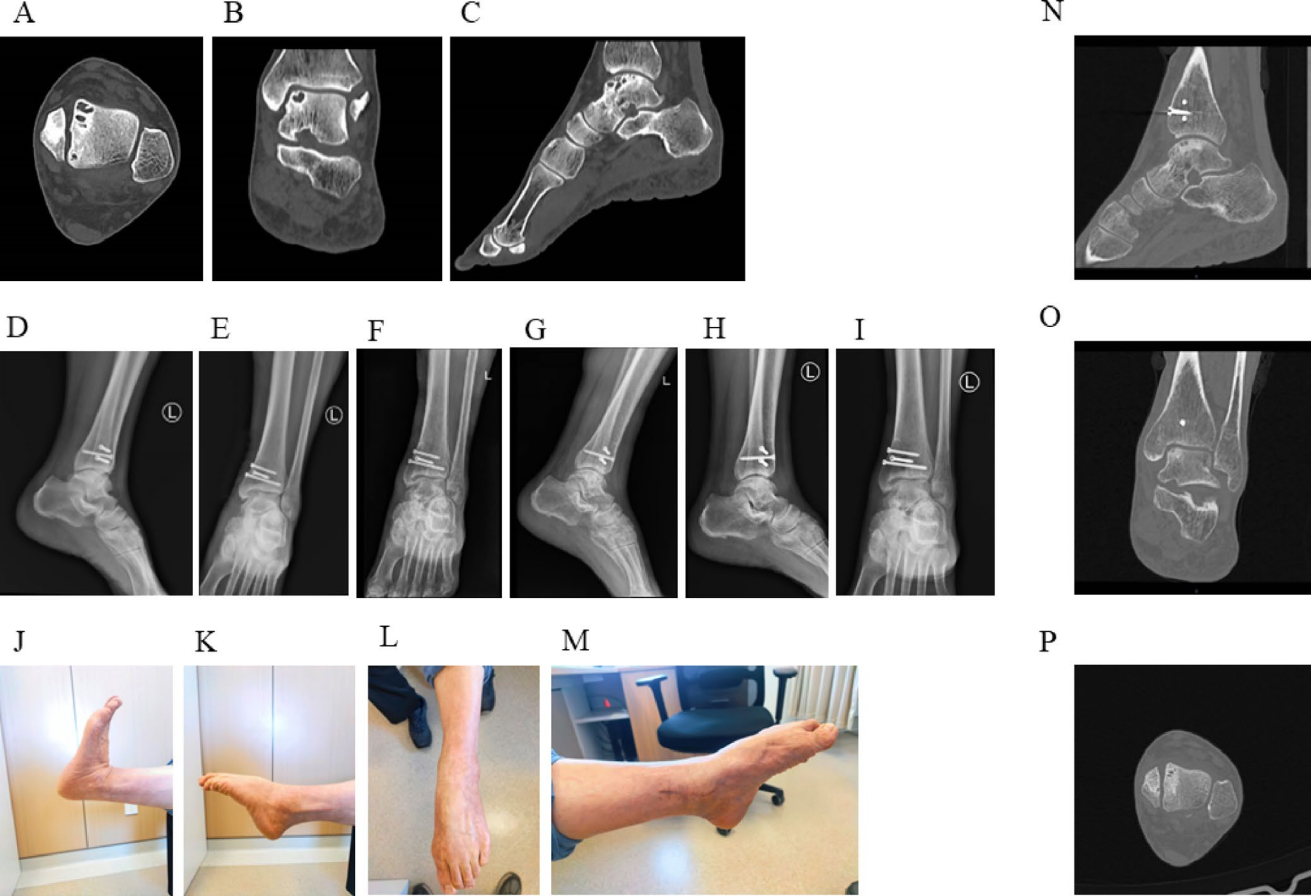
Images from another typical case of a large talar cyst. (A–C) Preoperative computed tomography (CT) images demonstrating a cystic lesion in the left talus—axial view (A), coronal view (B), and sagittal view (C); (D and E) lateral (D) and anteroposterior (E) radiographs of the left ankle on the second postoperative day showing satisfactory osteotomy reduction and screw placement; (F and G) radiographs at 3 months postoperatively showing indistinct osteotomy lines and early graft incorporation; (H and I) radiographs at 4 months postoperatively demonstrating progressive graft assimilation; (J–M) clinical photographs at 1 year postoperatively showing well‐healed surgical incisions and good functional recovery—dorsiflexion, lateral view (J), plantarflexion, lateral view (K), plantarflexion, frontal view (L), and plantarflexion, medial view (M); (N–P) Postoperative CT images at 16 months demonstrating complete fusion of the grafted bone with the surrounding talus and no evidence of cyst recurrence or degenerative changes—sagittal view (N), coronal view (O), and axial view (P).

A 43‐year‐old male patient was admitted to the hospital with “left foot pain for 1.5 years and a discovered left foot mass for 6 months.” The patient initially developed left foot pain of unknown etiology 1.5 years prior to admission. The pain was particularly pronounced during morning activities and worsened with physical activity. Over the subsequent 6 months, the pain progressively intensified, prompting him to visit our outpatient department. A CT examination revealed a “left talar cyst.” Preoperative imaging studies, including CT, confirmed the diagnosis (Figure 2A–C). The patient underwent medial malleolus triple‐plane osteotomy under general anesthesia. The talar cystic lesion was thoroughly debrided, and autologous iliac bone graft was harvested and implanted into the defect. The osteotomy site was anatomically reduced and fixed with three 4.0‐mm cannulated screws. The operation was uneventful, with primary wound healing and no neurovascular complications.

Postoperative radiographic follow‐up was conducted at multiple time points. Anteroposterior and lateral radiographs of the left ankle obtained on the second postoperative day confirmed satisfactory osteotomy reduction and screw placement (Figure 2D,E). At 3 months postoperatively, radiographs showed indistinct osteotomy lines and early incorporation of the graft (Figure 2F,G). By 4 months, further graft assimilation was observed (Figure 2H,I). At 1 year postoperatively, clinical photographs demonstrated well‐healed surgical incisions and good functional recovery of the left foot (Figure 2J–M). The patient returned for elective removal of internal fixation at 16 months postoperatively. CT examination at that time demonstrated complete fusion of the grafted bone with the surrounding talus, with no evidence of cyst recurrence or degenerative changes (Figure 2N–P).

At the most recent follow‐up (16 months postoperatively), the patient remained asymptomatic with full ankle range of motion. His AOFAS score improved from 58 preoperatively to 93 at final follow‐up, and VAS score decreased from 6 to 1.

## 4 Discussion

### 4.1 Summary of Main Findings

The present study evaluated the clinical and radiographic outcomes of medial malleolus triple‐plane osteotomy combined with autologous iliac bone grafting for the treatment of large talar cysts in nine patients. The procedure was successfully performed in all cases, with primary wound healing and no neurovascular injuries. Radiographic follow‐up demonstrated that osteotomy lines became indistinct at 3 months, progressively disappeared by 6 months, and were fully healed by 12 months, at which point internal fixation was removed. The grafted talar region exhibited sclerosis at 3 months, progressive assimilation with surrounding bone density at 6 months, and subtle visibility of the graft site at 12 months. Postoperative CT at 30 months for the representative case confirmed complete graft fusion. At final follow‐up (mean 23.0 ± 7.8 months), significant improvements were observed in VAS scores (from 6.3 ± 1.8 preoperatively to 2.4 ± 2.6 postoperatively, *p* < 0.05), AOFAS‐AHS scores (from 59.39 ± 6.31 to 92.37 ± 2.09, *p* < 0.05), and Kaikkonen functional scores (from 60.23 ± 2.79 to 89.11 ± 3.11, *p* < 0.05). These findings suggest that this combined approach is a safe and effective treatment option for large talar cysts.

### 4.2 Graft Selection

The nine cases in this study demonstrated similar characteristics to other populations with talar cysts, with a predominance in males around a mean age of 34.20 ± 9.23 years and a history of trauma (six of nine cases) [21]. Regarding graft selection, autologous iliac bone was chosen due to its well‐documented advantages. The iliac bone provides a substantial volume of graft material, making it suitable for large talar cysts regardless of lesion size. It offers an optimal balance of hardness and plasticity, providing both structural support and adaptability for shaping into various geometries. As an autologous graft, it eliminates risks of immune rejection or disease transmission and has a low incidence of donor site complications [1, 15]. In terms of recovery, patients in our series began toe‐touch weight‐bearing at 6 weeks and progressed to full weight‐bearing by 3 months postoperatively, with complete radiographic graft assimilation observed by 12 months.

Several other graft options exist for talar cyst reconstruction, each with distinct advantages and disadvantages:

Autologous osteochondral grafts (typically harvested from the ipsilateral knee) are commonly used for OLT repair. A comparison study by Shim et al. [22] suggested that autologous osteochondral transfer was superior in clinical improvements and survival rate in treating large talar cysts compared to bone marrow stimulation, which had an increased risk of failure. A meta‐analysis of 14 studies reported aggregate mean VAS improvement from 6.47 ± 1.35 preoperatively to 1.98 ± 1.18 postoperatively, and AOFAS improvement from 56.41 ± 8.52 to 87.14 ± 4.82 [23]. The advantages include hyaline cartilage coverage and structural integrity. However, disadvantages include limited graft availability, size mismatch with the recipient site, and donor site morbidity—Fraser et al. [11] reported that 12.5% of patients experienced knee symptoms postharvest, with complication rates ranging from 2% to 50%. Recovery time typically requires 6–8 weeks of nonweight‐bearing followed by gradual rehabilitation [23].

Allogeneic cartilage grafts eliminate donor site morbidity but face significant limitations including restricted donor availability, variable graft quality, risks of immune rejection, bone resorption, and economic constraints [12, 13]. Additionally, incorporation time may be prolonged compared to autografts, and long‐term outcomes remain less established [13].

Autogenous tibial periosteal bone grafting has shown promising results in a study of 36 cases, with significant improvements in pain and function scores [24]. Advantages include autologous tissue with osteogenic potential and a convenient harvest site within the same surgical field. However, the volume of available graft may be limited for larger cysts, and long‐term data remain limited [24].

Synthetic bone grafts with preserved cartilage flaps have been used in small series (*n* = 8), demonstrating improved VAS and AOFAS scores [25]. Advantages include unlimited availability and no donor site morbidity. Disadvantages include lack of osteogenic potential, slower incorporation, higher cost, and concerns about long‐term durability compared to autografts [25].

Talar osteo‐periostic grafting from the iliac crest, as reported by Dahmen et al. [26] in 43 patients, achieved 100% graft consolidation with improved function scores. This technique combines the advantages of iliac bone volume with periosteal osteogenic potential. Recovery outcomes showed all grafts consolidated, though detailed weight‐bearing protocols were similar to autograft techniques [26].

When comparing recovery parameters across graft types, most autograft techniques require 6–8 weeks of protected weight‐bearing followed by progressive rehabilitation, with radiographic evidence of graft integration typically observed between 3 and 12 months [23, 24, 25, 26]. Functional outcomes as measured by AOFAS scores generally improve from preoperative ranges of 43–59 to postoperative ranges of 82–93 across different graft types [23, 24, 25, 26, 27].

While direct comparison between our small series and larger studies is limited by sample size differences, our clinical improvements (VAS: from 6.3 ± 1.8 to 2.4 ± 2.6; AOFAS: from 59.39 ± 6.31 to 92.37 ± 2.09) appear comparable to outcomes reported for other autograft techniques. The absence of donor site complications in our series and the complete graft assimilation observed on follow‐up imaging support autologous iliac bone as a reliable option for large talar cysts. These collective findings indicate that multiple grafting approaches can effectively treat large talar cysts [27], with graft selection guided by lesion characteristics, donor site considerations, and surgeon experience.

### 4.3 Anatomical Rationale and Advantages of Triple‐Plane Osteotomy

Medial malleolar osteotomy is a common approach for exposing talar lesions. In this study, the triple‐plane osteotomy method was selected for lesion exposure because we considered it to have the following advantages: (1) *Wide exposure*: Triple‐plane osteotomy provides a broad surgical field, allowing precise and quantifiable osteotomy positioning. Additionally, the learning curve is short, facilitating mastery of the technique. (2) *Ease of reduction*: The technique allows for straightforward reduction and provides stable postoperative fixation with a low error rate during internal fixation. (3) *Improved healing*: The osteotomy creates three distinct planes of bone contact, resulting in a larger surface area conducive to rapid bone healing. (4) *Early functional recovery*: After fixation, the initial stability of the triple‐plane osteotomy enables early functional exercises of the ankle joint, promoting joint recovery, enhancing patient confidence, and increasing subjective satisfaction.

When selecting a medial malleolar osteotomy technique, minimizing damage to the cartilage of the distal tibial articular surface is crucial for preventing mid‐ to long‐term osteoarthritis. The anatomical study by Leumann et al. [28] clearly indicates that there is an area in the medial curvature of the distal tibia (the transition zone from the tibial plafond to the medial malleolus) with minimal cartilage coverage (< 75%). The subchondral bone plate in this area also has the lowest degree of mineralization and indentation strength, suggesting lower biomechanical loading. This study provides important anatomical evidence for the “ideal location” of a medial malleolar osteotomy. The design of the sagittal and coronal osteotomy lines in our triple‐plane osteotomy aims to precisely position the osteotomy surface within this area of low cartilage coverage. This theoretically minimizes secondary articular cartilage damage caused by the osteotomy, aligning with the current concept of optimizing osteotomy location based on anatomy to reduce long‐term complications.

### 4.4 Comparison With Other Osteotomy Techniques

The outcomes of our triple‐plane osteotomy compare favorably with those reported for other medial malleolar osteotomy techniques [29, 30, 31, 32], as summarized in Table 1. The retrospective study by Bull et al. [29] showed that bi‐plane chevron medial malleolar osteotomy fixed with 2 lag screws had a postoperative malunion rate as high as 30%, with an average displacement of about 2 mm. Furthermore, 26% of patients required reoperation for hardware removal due to a related issue. The study attributed this high complication rate to shear stress generated by a mismatch between the screw angle and the osteotomy plane, suggesting the addition of a third transverse screw or a buttress plate to enhance stability [29].

**TABLE 1:** Comparison of different medial malleolar osteotomy techniques.

Osteotomy type	Surgical technique	Advantages	Disadvantages/complications
Oblique osteotomy	Single oblique cut through the medial malleolus	Excellent dorsal exposure of the talus; allows easy compression across osteotomy with minimal deltoid dissection	High rates of osteoarthritis (20%–50% within 5 years); delayed union and nonunion in 7.9%
Bi‐pchevron osteotomy	Inverted V‐shaped cut with apex in medial distal tibial midline	Perpendicular instrumentation access to medial talus; wide exposure; deltoid ligament preservation; large cancellous surface area for healing	Malunion rate as high as 30% with 2‐screw fixation; average displacement 1.8 mm proximal and 1.2 mm medial; 26% require reoperation for hardware removal
Triple‐plane osteotomy (current study)	Three mutually perpendicular cuts (sagittal, coronal, horizontal planes)	Broad surgical field; precise positioning; three‐plane bone contact creates mechanical interlocking; allows multiscrew vertical compression; preserves posteromedial structures; early functional recovery (average ~8 weeks to weight‐bearing)	No malunion or nonunion in this series; requires precise surgical technique; limited long‐term data beyond small series

Although the oblique osteotomy is widely employed, historical reports [30, 31, 32] indicate that it is associated with a high incidence of osteoarthritis, as well as complications such as delayed union and nonunion. McCullough and Venugopal [30] reported osteoarthritic changes in 20% of cases within 5 years after osteotomy, while Gaulrapp et al. [31] reported osteoarthritic changes in 50% of cases. Lee et al. [32] reported delayed union and nonunion in 7.9% of patients.

Compared with the 30% malunion rate reported by Bull et al. [29], the outcome in our (albeit small) series suggests a potential advantage of the triple‐plane osteotomy in maintaining reduction stability.

The triple‐plane osteotomy creates three mutually perpendicular bone contact surfaces: sagittal, coronal, and horizontal. This not only provides a larger surface area for bone healing, but its geometric shape also creates a mechanical interlocking effect similar to a “mortise and tenon joint,” effectively resisting rotational and shear stresses. This allows for the use of multiple (typically 3) cannulated screws for vertical compression fixation in different planes, achieving excellent initial stability. This stability is the foundation for allowing patients to start early (average ~8 weeks in this study) progressive weight‐bearing functional exercises and may reduce the risk of postoperative displacement due to inadequate fixation, as seen in the study by Bull et al. [29].

As described in the technique, the osteotomy block can be completely flipped open, providing a broad and direct view of the posteromedial talar lesion, facilitating precise lesion management and graft implantation. Simultaneously, the regular multiplane osteotomy surfaces make the anatomical reduction of the osteotomy block more intuitive and precise during surgery, aiding in the restoration of joint surface congruity and avoiding joint incongruity due to malreduction. The latter has been confirmed by the literature as a significant cause of local degeneration and pain [28, 29].

The osteotomy protocol has clear steps, and the osteotomy lines are easy to locate and complete, facilitating mastery by surgeons. Furthermore, the design preserves the continuity of the posteromedial bone of the medial malleolus, helping to protect the posterior tendons and neurovascular bundle.

## 5 Limitations

This study has some limitations, as a small case series in one clinical center, the number of patients was limited and there was no comparison group. The small sample size and retrospective design limit the strength of our conclusions. Our study is descriptive and hypothesis‐generating rather than comparative or definitive. Therefore, we cannot presume that this approach is more effective than any other. Larger prospective studies are needed to evaluate whether this method of treating large talar cysts is superior to alternative methods. Graft fusion was assessed radiographically via X‐ray and that CT would provide more detailed evaluation of bony integration. Furthermore, future studies should incorporate systematic CT follow‐up to collect more detailed data on bony integration, thereby ensuring more reliable prognostic assessments.

Despite these limitations, the triple‐plane osteotomy technique described herein has promising prospects for clinical application. Its anatomical rationale positioning the osteotomy within the area of minimal cartilage coverage as identified by Leumann et al. [28] theoretically reduces the risk of iatrogenic cartilage damage and long‐term osteoarthritis. The biomechanical advantages of three‐plane interlocking fixation provide excellent initial stability, enabling early functional rehabilitation and potentially reducing malunion rates compared to conventional techniques [29]. The procedure's clear and reproducible surgical steps facilitate mastery by surgeons, suggesting good potential for broader adoption in specialized foot and ankle centers. Future multicenter prospective studies with standardized protocols, larger sample sizes, longer follow‐up periods, and systematic CT evaluation are warranted to validate these preliminary findings and establish the role of this technique in the treatment algorithm for large talar cysts.

## 6 Conclusion

In summary, the treatment of talar cysts using triple‐plane medial malleolar osteotomy combined with autologous iliac bone grafting demonstrated favorable clinical results in this small case series and appears to be a feasible and promising technique. Further prospective comparative studies are warranted to evaluate its role relative to other established methods.

## Author Contributions

**Yanbin Teng** and **Xiaoming Yang** carried out the studies, participated in data collection, and drafted the manuscript. **Yan Zhang**, **Maoyuan Xin**, **Longxin An**, and **Jie Zhao** performed the statistical analysis and participated in its design. Yanbin Teng and Xiaoming Yang participated in the acquisition, analysis, and interpretation of data, and revised the manuscript. All authors read and approved the final manuscript.

## Funding

The authors have nothing to report.

## Disclosure

(i) That all authors listed meet the authorship criteria according to the latest guidelines of the International Committee of Medical Journal Editors, and (ii) that all authors are in agreement with the manuscript.

## Ethics Statement

This study was approved by the Ethics Committee of Weifang People's Hospital (KYLL20241220‐2), and informed consent was obtained from all participants. I confirm that all methods were performed in accordance with the relevant guidelines. All procedures were performed in accordance with the ethical standards laid down in the 1964 Declaration of Helsinki and its later amendments.

## Conflicts of Interest

The authors declare no conflicts of interest.

## Supporting information


**Figure S1:** os70308‐sup‐0001‐FigureS1.tif.

## Data Availability

The data that supports the findings of this study are available in the Supporting Information of this article.

## References

[1] J. Bruns , C. Habermann , and M. Werner , “Osteochondral Lesions of the Talus: A Review on Talus Osteochondral Injuries, Including Osteochondritis Dissecans,” Cartilage 13 (2021): 1380s–1401s. 33423507 10.1177/1947603520985182 PMC8808845

[2] U. Brulc , M. Drobnič , M. Kolar , and K. Stražar , “A Prospective, Single‐Center Study Following Operative Treatment for Osteochondral Lesions of the Talus,” Foot and Ankle Surgery 28 (2022): 714–719. 34518042 10.1016/j.fas.2021.08.008

[3] Q. G. H. Rikken and G. Kerkhoffs , “Osteochondral Lesions of the Talus: An Individualized Treatment Paradigm From the Amsterdam Perspective,” Foot and Ankle Clinics 26 (2021): 121–136. 33487235 10.1016/j.fcl.2020.10.002

[4] J. R. Steele , T. J. Dekker , A. E. Federer , J. L. Liles , S. B. Adams , and M. E. Easley , “Republication of “Osteochondral Lesions of the Talus: Current Concepts in Diagnosis and Treatment”,” Foot & Ankle Orthopaedics 8 (2023): 24730114231192961. 37566685 10.1177/24730114231192961 PMC10408332

[5] M. E. Dombrowski , Y. Yasui , C. D. Murawski , et al., “Conservative Management and Biological Treatment Strategies: Proceedings of the International Consensus Meeting on Cartilage Repair of the Ankle,” Foot & Ankle International 39 (2018): 9s–15s. 30215314 10.1177/1071100718779390

[6] J. Wolfe , B. Derner , and R. T. Scott , “Management of Subchondral Lesions in the Foot and Ankle,” Clinics in Podiatric Medicine and Surgery 40 (2023): 553–568. 37236691 10.1016/j.cpm.2023.03.005

[7] Y. K. Lee , K. W. Young , J. S. Kim , H. S. Lee , W. J. Cho , and H. N. Kim , “Arthroscopic Microfracture With Atelocollagen Augmentation for Osteochondral Lesion of the Talus: A Multicenter Randomized Controlled Trial,” BMC Musculoskeletal Disorders 21 (2020): 716. 33143647 10.1186/s12891-020-03730-3 PMC7640454

[8] C. C. H. Li and T. H. Lui , “Management of Bone Cyst of Talar Body by Endoscopic Curettage, Nanofracture, and Bone Graft Substitute,” Arthroscopy Techniques 10 (2021): e1985–e1993. 34401244 10.1016/j.eats.2021.04.026 PMC8355510

[9] R. T. Powers , T. C. Dowd , and E. Giza , “Surgical Treatment for Osteochondral Lesions of the Talus,” Arthroscopy 37 (2021): 3393–3396. 34863377 10.1016/j.arthro.2021.10.002

[10] F. Krause and H. Anwander , “Osteochondral Lesion of the Talus: Still a Problem?,” EFORT Open Reviews 7 (2022): 337–343. 35638600 10.1530/EOR-22-0024 PMC9257727

[11] E. J. Fraser , M. C. Harris , M. P. Prado , and J. G. Kennedy , “Autologous Osteochondral Transplantation for Osteochondral Lesions of the Talus in an Athletic Population,” Knee Surgery, Sports Traumatology, Arthroscopy 24 (2016): 1272–1279. 10.1007/s00167-015-3606-8 25962962

[12] V. Chopra , D. Chang , A. Ng , D. L. Kruse , and P. A. Stone , “Arthroscopic Treatment of Osteochondral Lesions of the Talus Utilizing Juvenile Particulated Cartilage Allograft: A Case Series,” Journal of Foot and Ankle Surgery 59 (2020): 436–439. 10.1053/j.jfas.2019.09.005 32131018

[13] J. E. Manzi , K. Manchanda , M. H. Nasra , et al., “Long‐Term Patient Outcomes for Treatment of Difficult Osteochondral Lesions of the Talus With Particulated Juvenile Allograft Cartilage Implantation ± Calcaneal Autograft: A Cohort Study,” European Journal of Orthopaedic Surgery and Traumatology 34 (2024): 561–568. 37650974 10.1007/s00590-023-03642-7

[14] F. Migliorini , F. Cuozzo , E. Torsiello , F. Spiezia , F. Oliva , and N. Maffulli , “Autologous Bone Grafting in Trauma and Orthopaedic Surgery: An Evidence‐Based Narrative Review,” Journal of Clinical Medicine 10 (2021): 4347. 34640364 10.3390/jcm10194347 PMC8509778

[15] W. Shi , S. Yang , S. Xiong , et al., “Comparison of Autologous Osteoperiosteal and Osteochondral Transplantation for the Treatment of Large, Medial Cystic Osteochondral Lesions of the Talus,” American Journal of Sports Medicine 50 (2022): 769–777. 35048728 10.1177/03635465211068529

[16] L. Ramponi , Y. Yasui , C. D. Murawski , et al., “Lesion Size Is a Predictor of Clinical Outcomes After Bone Marrow Stimulation for Osteochondral Lesions of the Talus: A Systematic Review,” American Journal of Sports Medicine 45 (2017): 1698–1705. 27852595 10.1177/0363546516668292

[17] P. C. Kreuz , M. Steinwachs , M. Edlich , et al., “The Anterior Approach for the Treatment of Posterior Osteochondral Lesions of the Talus: Comparison of Different Surgical Techniques,” Archives of Orthopaedic and Trauma Surgery 126 (2006): 241–246. 16273380 10.1007/s00402-005-0058-5

[18] J. Dahmen , K. T. A. Lambers , M. L. Reilingh , C. J. A. van Bergen , S. A. S. Stufkens , and G. Kerkhoffs , “No Superior Treatment for Primary Osteochondral Defects of the Talus,” Knee Surgery, Sports Traumatology, Arthroscopy 26 (2018): 2142–2157. 10.1007/s00167-017-4616-5 PMC6061466 28656457

[19] H. B. Kitaoka , I. J. Alexander , R. S. Adelaar , J. A. Nunley , M. S. Myerson , and M. Sanders , “Clinical Rating Systems for the Ankle‐Hindfoot, Midfoot, Hallux, and Lesser Toes,” Foot & Ankle International 15 (1994): 349–353. 7951968 10.1177/107110079401500701

[20] G. Z. Heller , M. Manuguerra , and R. Chow , “How to Analyze the Visual Analogue Scale: Myths, Truths and Clinical Relevance,” Scandinavian Journal of Pain 13 (2016): 67–75. 28850536 10.1016/j.sjpain.2016.06.012

[21] P. R. van Diepen , J. Dahmen , J. N. Altink , S. A. S. Stufkens , and G. Kerkhoffs , “Location Distribution of 2,087 Osteochondral Lesions of the Talus,” Cartilage 13 (2021): 1344s–1353s. 32909458 10.1177/1947603520954510 PMC8808869

[22] D. W. Shim , K. H. Park , J. W. Lee , Y. J. Yang , J. Shin , and S. H. Han , “Primary Autologous Osteochondral Transfer Shows Superior Long‐Term Outcome and Survival Rate Compared With Bone Marrow Stimulation for Large Cystic Osteochondral Lesion of Talus,” Arthroscopy 37 (2021): 989–997. 33276050 10.1016/j.arthro.2020.11.038

[23] K. M. Feeney , “The Effectiveness of Osteochondral Autograft Transfer in the Management of Osteochondral Lesions of the Talus: A Systematic Review and Meta‐Analysis,” Cureus 14 (2022): e31337. 36514582 10.7759/cureus.31337 PMC9741491

[24] S. Cao , W. Ji , Q. Zan , et al., “Evaluation of the Efficacy of Autogenous Tibial Periosteal Bone Grafting in the Treatment of Osteochondral Lesions of the Talus and Analysis of Three‐Dimensional Factors in the Necrotic Zone,” International Orthopaedics 48 (2024): 1831–1838. 38558192 10.1007/s00264-024-06161-0

[25] X. Yao , Z. Yun , Y. Du , X. Xie , S. Chen , and X. Cheng , “Synthetic Bone Grafting With Preserved Cartilage Flap via a Medial Malleolus Osteotomy Approach to Treat Osteochondral Lesion of the Talus: Technical Note and Preliminary Clinical Results,” International Orthopaedics 47 (2023): 2743–2749. 37548695 10.1007/s00264-023-05920-9

[26] J. Dahmen , Q. Rikken , S. A. S. Stufkens , and G. Kerkhoffs , “Talar OsteoPeriostic Grafting From the Iliac Crest (TOPIC): Two‐Year Prospective Results of a Novel Press‐Fit Surgical Technique for Large, Complex Osteochondral Lesions of the Medial Talus,” Journal of Bone and Joint Surgery. American Volume 105 (2023): 1318–1328. 37363948 10.2106/JBJS.22.01322

[27] L. Cheng and X. Wang , “Advancements in the Treatment of Osteochondral Lesions of the Talus,” Journal of Orthopaedic Surgery and Research 19 (2024): 827. 39639331 10.1186/s13018-024-05314-6 PMC11622651

[28] A. Leumann , M. Horisberger , O. Buettner , M. Mueller‐Gerbl , and V. Valderrabano , “Medial Malleolar Osteotomy for the Treatment of Talar Osteochondral Lesions: Anatomical and Morbidity Considerations,” Knee Surgery, Sports Traumatology, Arthroscopy 24 (2016): 2133–2139. 10.1007/s00167-015-3591-y 25854498

[29] P. E. Bull , G. C. Berlet , C. Canini , and C. F. Hyer , “Rate of Malunion Following bi‐Plane Chevron Medial Malleolar Osteotomy,” Foot & Ankle International 37 (2016): 620–626. 26843546 10.1177/1071100716628912

[30] C. J. McCullough and V. Venugopal , “Osteochondritis Dissecans of the Talus: The Natural History,” Clinical Orthopaedics and Related Research 144 (1979): 264–268. 535235

[31] H. Gaulrapp , F. W. Hagena , and G. Wasmer , “Postoperative Evaluation of Osteochondrosis Dissecans of the Talus With Special Reference to Medial Malleolar Osteotomy,” Zeitschrift für Orthopädie und Ihre Grenzgebiete 134 (1996): 346–353. 8928564 10.1055/s-2008-1039773

[32] K. T. Lee , J. S. Kim , K. W. Young , et al., “The Use of Fibrin Matrix‐Mixed Gel‐Type Autologous Chondrocyte Implantation in the Treatment for Osteochondral Lesions of the Talus,” Knee Surgery, Sports Traumatology, Arthroscopy 21 (2013): 1251–1260. 10.1007/s00167-012-2096-1 PMC3657090 22752415

