# Supplementary figures and images for: Medial Malleolus Triple‐Plane Osteotomy With Autologous Iliac Bone Grafting for the Treatment of Talar Cysts
Source: Orthop Surg. 2026 Apr 9;18(5):1075–84. doi: 10.1111/os.70308 (PMC13138908; doi:10.1111/os.70308)

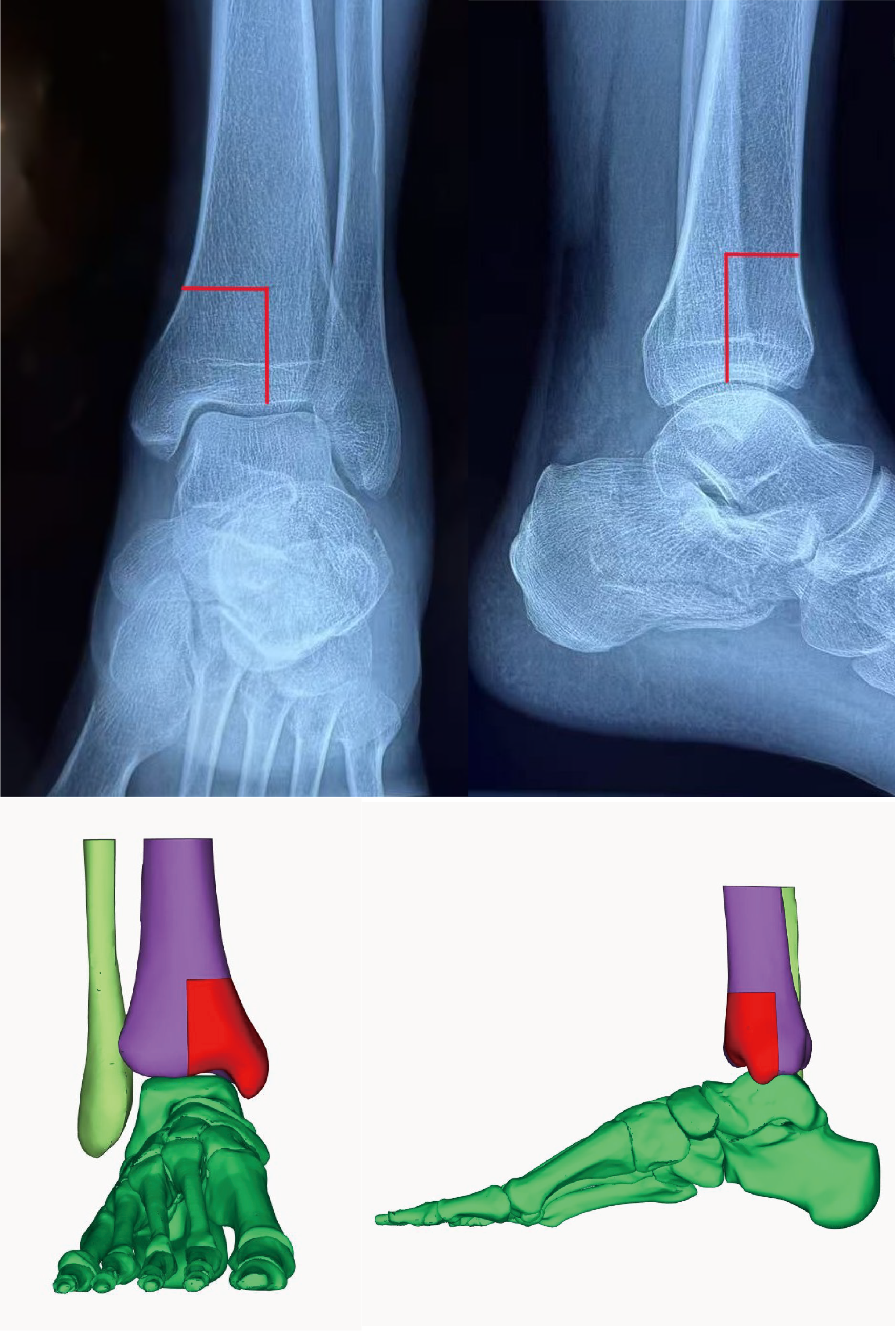

Supplement: Supplementary file 1 — Figure S1: os70308‐sup‐0001‐FigureS1.tif. [file OS-18-1075-s001.tif]
